# Effect of Surface Tension of Cleaning Solutions on the Cleanability of Through-Glass via (TGV) Substrates †

**DOI:** 10.3390/mi17080920

**Published:** 2026-07-30

**Authors:** Hiroaki Nishiza, Masahito Horie, Masakazu Ito, Hisao Enomoto

**Affiliations:** Research and Development Division, Parker Corporation, Tokyo 135-0051, Japan

**Keywords:** through-glass via, TGV, glass substrate, wet cleaning, surface tension, surfactant, cleanability, electroless copper plating, cleaner

## Abstract

Through-glass via (TGV) substrates have attracted increasing attention as interposers and core substrates for advanced packaging. However, contaminants adhere to glass surfaces; in particular, airborne organic substances increase the contact angle, hindering the penetration of cleaning solutions into TGVs and thereby reducing the cleaning effect. Reducing the cleaning-solution surface tension by adding a surfactant may promote penetration into TGVs with high contact angles. This study investigated the effect of cleaning-solution surface tension on cleanability inside TGVs. Under the tested conditions, the contact angle outside the TGV after cleaning was 3–4° for all cleaning solutions, regardless of their surface tension. In contrast, the contact angle inside the TGV after cleaning decreased with decreasing surface tension. At 27.5 and 41.8 mN/m, the contact angle inside the TGV after cleaning decreased to 3–4° under both conditions. These results indicate that the surface tension of the cleaning solution affects the cleanability inside the TGV. For a TGV with an opening diameter of 20 μm and an aspect ratio of 10, cleaning with a 27.5 mN/m solution prior to electroless Cu plating was associated with reduced void formation and film thickness variation in the Cu film inside the TGV under the tested conditions.

## 1. Introduction

In recent years, glass has attracted increasing attention as a material for interposers and package core substrates in three-dimensional packaging and chiplet integration. In these applications, glass substrates incorporating through-hole structures called through-glass via (TGV), with diameters ranging from several tens to 100 μm, are used (hereinafter referred to as TGV glass). Producing glass in large-area panels enables cost reduction. Moreover, glass exhibits excellent electrical properties, high surface flatness, and a coefficient of thermal expansion close to that of Si, making it a promising next-generation material [1,2,3,4,5,6,7,8,9,10].

However, during processing, packaging, transportation, and storage, glass surfaces can become contaminated with glass cullet, residues transferred from protective cushioning materials, cutting fluids, fingerprints, and particles [11,12,13]. Organic substances in the atmosphere can adsorb onto the glass surface causing the contact angle of the originally hydrophilic glass surface to increase over time (referred to as hydrophobization) [14,15]. Such contamination can degrade film adhesion and induce defects such as pinholes [16,17,18,19]. Therefore, for TGV glass, for which Cu film formation by plating is being actively investigated, removal of surface contaminants through cleaning is essential to ensure consistent quality [20,21,22,23,24,25,26,27].

Cleaning methods for glass substrates are generally classified into dry cleaning (such as plasma, laser, and thermal decomposition) and wet cleaning (using chemical cleaning solutions). Dry cleaning requires expensive equipment and is associated with challenges such as significant damage to the glass surface and limitations in removing inorganic particulates and glass cullet. In contrast, wet cleaning is relatively inexpensive, and because it is highly effective against various organic and inorganic contaminants and has minimal impact on the glass surface, it has been widely used for cleaning glass substrates [28,29,30,31]. Most conventional studies on glass cleaning have targeted bare glass; glass with fine through-holes, such as TGV glass, has not been thoroughly investigated [32,33,34,35]. Bowman et al. reported that as glass surfaces inside capillary tubes made of fused silica, borosilicate glass, and lead glass become hydrophobized due to contamination, it becomes difficult for water to penetrate the capillaries [36]. This is because capillary pressure decreases with increasing contact angle of the glass surface inside the capillaries due to contamination [37]. In other words, for TGV glass where hydrophobization has progressed due to contamination, as the contact angle of the glass surface inside the TGV (hereinafter referred to as “inside the TGV”) also increases, the cleaning solution may not easily penetrate the TGV interior, making cleaning difficult. Therefore, as TGV glass, unlike regular bare glass, requires cleaning not only the glass surface outside the TGV (hereinafter referred to as “outside the TGV”) but also inside it, a method for efficient cleaning that enables the cleaning solution to penetrate the hydrophobized TGV interior is required.

The addition of a surfactant to the cleaning solution to reduce the surface tension and improve wettability is considered an effective approach to promote penetration of the solution into the hydrophobized TGV interior. Surfactants have the effect of lowering the surface tension of liquids [38,39]. Young’s equation, which represents the relationship between surface tension and contact angle at equilibrium, is shown in Equation (1).

(1) $$\cos\theta =\frac{\gamma_{sv}-\gamma_{sl}}{\gamma_{lv}}$$

Here, *θ* is the contact angle, *γ_sv_* is the surface free energy of the solid surface, *γ_sl_* is the interfacial tension between the solid and liquid, and *γ_lv_* is the surface tension of the liquid. From Equation (1), adding a surfactant to the cleaning solution lowers *γ_lv_*, and as adsorption of the surfactant at the solid–liquid interface lowers *γ_sl_*, *θ* is prone to decrease. Therefore, a cleaning solution with improved wettability via surfactant addition can easily wet hydrophobic surfaces [40]. Next, as TGVs are fine through-holes, capillary pressure is considered to affect the penetration of liquid into the TGV interior. The equation for capillary pressure is shown in Equation (2).

(2) $$P_{C}=\frac{2\sigma \cos\theta }{r}$$

Here, *P_c_* is the capillary pressure, *σ* is the surface tension of the liquid, *θ* is the contact angle, and *r* is the capillary radius. Note that the surface tension of water at 25 °C is approximately 72 mN/m. For a system where *σ* and *r* are fixed, *P_c_* increases as *θ* approaches 0°. That is, as clean glass is hydrophilic and exhibits a *θ* value close to 0°, water, which has a high *σ*, results in a high *P_c_* and spontaneously penetrates the TGV interior. In contrast, when a glass surface is contaminated, as *θ* increases, *P_c_* decreases. In particular, when *θ* exceeds 90°, as *P_c_* is negative, water does not spontaneously penetrate the TGV interior [41]. Accordingly, adding a surfactant to the cleaning solution to improve wettability is considered to enable the cleaning solution to penetrate the hydrophobized TGV interior. Notably, adding a surfactant decreases *σ* with a decrease in *θ*. Therefore, in the case of a surface state where the *θ* of water is less than 90°, *P_c_* may become lower than that of water, which has a high *σ*. However, it has been reported that Equation (2) cannot be simply applied to the penetration phenomenon of systems containing surfactants. This is because dynamic wetting is promoted by the repeated temporal variation in *σ* caused by the behavior of surfactant molecules. Thus, in surfactant-containing systems, penetration driven by capillary pressure may be promoted through the synergistic effects of a reduced *θ* and enhanced dynamic wetting associated with surfactant molecular behavior [42,43,44,45]. The addition of a surfactant to lower the surface tension of the cleaning solution may promote the penetration of the cleaning solution into the hydrophobized TGV interior and is expected to contribute to efficient cleaning inside the TGV.

Based on these ideas, we compared an alkaline cleaning solution whose surface tension was reduced to 27.5 mN/m by adding a surfactant (hereinafter referred to as the developed product) with a NaOH aqueous solution having a surface tension of 74.4 mN/m. The comparison focused on the cleanability inside the TGV with an opening diameter of *φ*60 μm and an aspect ratio (AR) of 6.7, the time required for the cleaning solution to penetrate the TGV interior, and the effect of cleaning on the electroless Cu plating film. The developed product markedly shortened the penetration time and lowered the contact angle inside the TGV. Hereinafter, the ability of the cleaning solution to lower the contact angle on the glass surface is referred to as “cleanability”. Note that a lower contact angle after cleaning indicates better cleanability of the cleaning solution. Furthermore, when electroless Cu plating was performed on the TGV glass under each cleaning condition, voids were formed in the electroless Cu film (hereinafter referred to as Cu film) inside the TGV under the uncleaned condition and when cleaned with the NaOH aqueous solution. In comparison, cleaning with the developed product did not result in voids in the Cu film. In addition, void formation in the Cu film inside the TGV decreased as the contact angle after cleaning decreased. These results suggest that the surface tension of the cleaning solution affects the cleanability inside the TGV [46]. However, the relationship between the surface tension of the cleaning solution and the cleanability inside the TGV has not been systematically clarified.

In this study, to systematically examine the relationship between cleaning-solution surface tension and the cleanability of TGV glass, we evaluated the effect of cleaning-solution surface tension on the cleanability of TGV glass using cleaning solutions with surface tensions ranging from 27.5 to 74.4 mN/m. Furthermore, the influence of cleaning on the electroless Cu plating treatment was investigated using TGV glass with an opening diameter of *φ*20 μm and an AR of 10, which had a smaller opening diameter and a higher AR than the TGV glass used in the previous study.

## 2. Materials and Methods

### 2.1. Substrate

**Figure 1:**
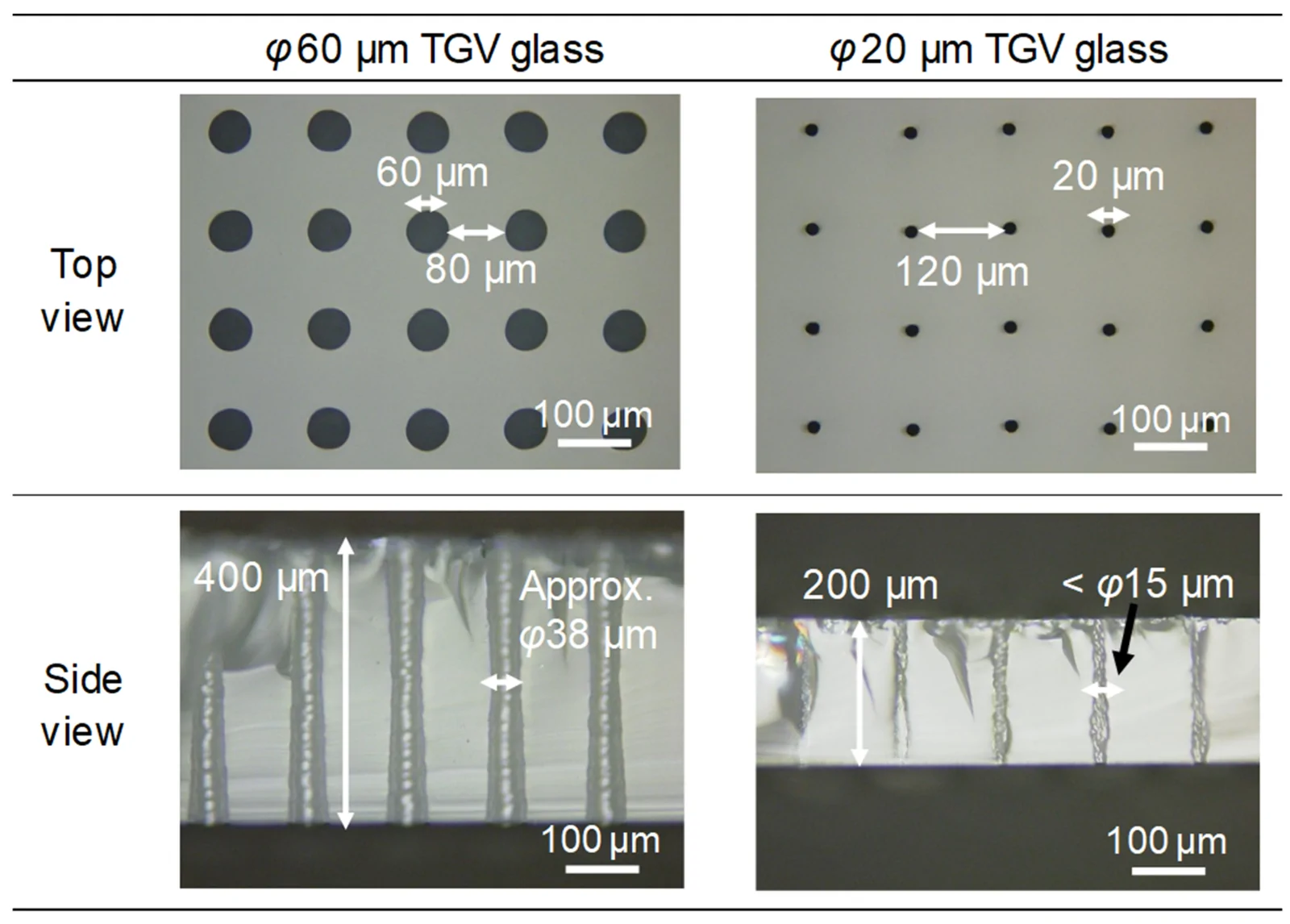
TGV shapes of the *φ*60 μm glass and *φ*20 μm glass used in the tests.

Alkali-free glass (Eagle XG, Corning Inc., Corning, NY, USA) was used as the glass substrate, and glass substrates with prefabricated TGVs were purchased from NSC Co., Ltd. (Toyonaka, Osaka, Japan). Two types of TGVs were used: one with an opening diameter of *φ*60 μm (0.4 mm × 25 mm × 75 mm, AR 6.7) (hereinafter referred to as *φ*60 μm glass) and one with an opening diameter of *φ*20 μm (0.2 mm × 25 mm × 75 mm, AR 10) (hereinafter referred to as *φ*20 μm glass). The TGV is hourglass-shaped. The detailed conditions used for TGV fabrication were not disclosed by the supplier (NSC Co., Ltd.). Figure 1 shows the TGV shapes of the *φ*60 μm glass and *φ*20 μm glass used in the tests. For the received TGV glass, the surface protective film was removed immediately before the tests, and the glass was used without further pretreatment.

### 2.2. Cleaning Solution

For the cleaning solutions, a 5 wt% aqueous NaOH solution (hereinafter referred to as NaOH solution) (surface tension: 74.4 mN/m) and alkaline cleaning solutions adjusted to surface tensions of 27.5 mN/m (developed product), 41.8 mN/m (hereinafter referred to as ST40), 51.9 mN/m (hereinafter referred to as ST50), and 58.4 mN/m (hereinafter referred to as ST60) by adding surfactants were used. The NaOH solution was prepared by adding NaOH as the alkaline component to deionized water and stirring for 30 min. The developed product, ST40, ST50, and ST60 were prepared by adding KOH as the alkaline component to deionized water, followed by the addition of surfactants and additives, and stirring for 30 min. The surfactants consisted of a mixture of commercially available nonionic and anionic surfactants and were added at concentrations above their respective critical micelle concentrations (CMC). Table 1 presents the components and properties of the cleaning solutions used in the tests. The surface tension of each cleaning solution was measured using the Wilhelmy method with a CBVP-Z (Kyowa Interface Science Co., Ltd., Niiza, Saitama, Japan) after adjusting the solution temperature to 25 °C.

### 2.3. Cleaning Test Method

The *φ*60 μm glass was immersed in each cleaning solution heated to 50 °C, and ultrasonic cleaning was performed at 38 kHz and 200 W for 1 min. Subsequently, the glass was rinsed with deionized water at a flow rate of approximately 4 L/min for 1 min, thereby replacing the cleaning solution with water and removing its components. Water droplets were then removed by air blowing, and the contact angles outside and inside the TGV were measured to evaluate the cleanability of the TGV substrate. Although contact angle measurement is an indirect method for evaluating cleanability and does not directly assess residual contaminants, it enables simple and rapid evaluation of cleanability. The contact angle outside the TGV was measured using a CA-150X (Kyowa Interface Science Co., Ltd.). The contact angle inside the TGV was measured using a micro-contact angle meter (MCA-J2, Kyowa Interface Science Co., Ltd.) after exposing the inner wall of the TGV by cutting the glass at the center of the TGV using a glass cutter. A GCC-P-M17P glass cutter (Mitsuboshi Diamond Industrial Co., Ltd., Settsu, Osaka, Japan) was used. To prevent contamination of the glass, the glass cutter was used without oil. For cutting, a score line was made on the glass surface along the intended cutting line using the glass cutter, and the glass was then carefully broken along the score line by applying stress. Because glass cullet generated during cutting could affect the contact angle measurement, it was promptly removed by air blowing. As the contact angle on the glass surface increases over time, it was measured promptly after the cleaning treatment. The contact angle measurement was performed five times, and the average value was calculated.

### 2.4. Electroless Copper Plating Test Method

**Figure 2:**
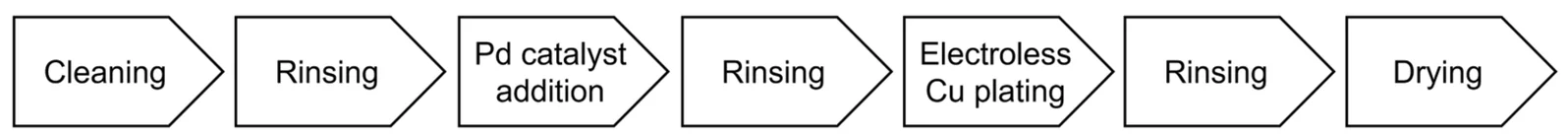
Schematic of the electroless Cu plating process of the *φ*20 μm glass.

The *φ*20 μm glass was immersed in the developed product heated to 50 °C, and ultrasonic cleaning was performed at 38 kHz and 200 W for 1 min. After an overflow rinse with deionized water at room temperature for 1 min, a Pd catalyst was applied, and electroless Cu plating was performed. No surface roughening treatment or adhesion-layer formation was performed on the glass surface. The electroless Cu plating treatment was outsourced to Koto Electric Co., Ltd. (Kawaguchi, Saitama, Japan). Figure 2 shows a schematic of the electroless Cu plating test process. Table 2 presents the electroless Cu plating process of the *φ*20 μm glass. The electroless Cu plating conditions listed in Table 2 were provided by Koto Electric Co., Ltd.; other detailed conditions were not disclosed by the contractor. Next, after resin embedding, the TGV glass subjected to electroless Cu plating was roughly sectioned using a DAD3220 dicing saw (Disco Corporation, Ota, Tokyo, Japan). Subsequently, the milling position was adjusted using the microscope attached to the ion milling system, and a TGV cross section was prepared using the ion milling system (JEOL Ltd., Akishima, Tokyo, Japan; model undisclosed). An Ar ion beam was used for ion milling, with an accelerating voltage of 6 kV, an irradiation direction of 90°, and a milling time of approximately 4–5 h. In addition, the sample was oscillated during milling. Mechanical polishing, sample cooling, and finishing at a low accelerating voltage were not performed. After preparation of the TGV cross section, the sample surface was coated with osmium, and the interface between the glass and Cu film (hereinafter referred to as the glass/Cu interface) was observed by SEM using a ULTRA55 (Carl Zeiss Microscopy GmbH, Oberkochen, Germany) at an accelerating voltage of 2 kV. The preparation of the TGV cross sections and SEM observations was outsourced to the Foundation for Promotion of Material Science and Technology of Japan (MST; Setagaya, Tokyo, Japan). For the uncleaned condition, the test was started from the application of the Pd catalyst. The experiment was performed once for each condition.

### 2.5. Measurement Method of Cleaning Solution Penetration Time into φ20 μm Glass TGV

**Figure 3:**
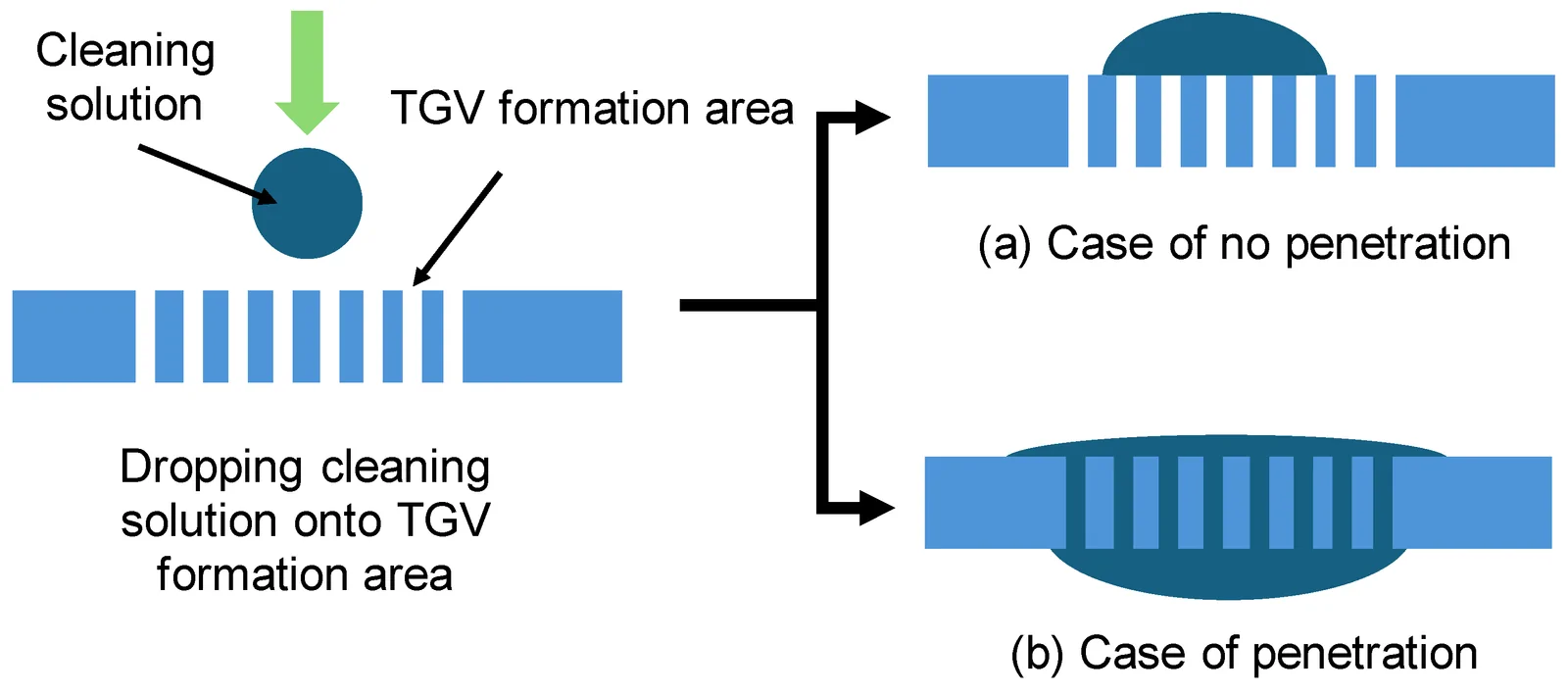
Schematic diagram of the method of dropping the cleaning solution onto the TGV formation area of the *φ*20 μm glass.

The cleaning solution was dropped onto the TGV formation area of the *φ*20 μm glass, and the time required for the cleaning solution to penetrate to the back side was measured. The experiment was performed three times for each condition, and the average value was calculated. Figure 3 shows the schematic of the method of dropping the cleaning solution onto the TGV formation area of the *φ*20 μm glass.

## 3. Results and Discussion

### 3.1. Effect of Surface Tension on the Cleanability of TGV Glass

**Figure 4:**
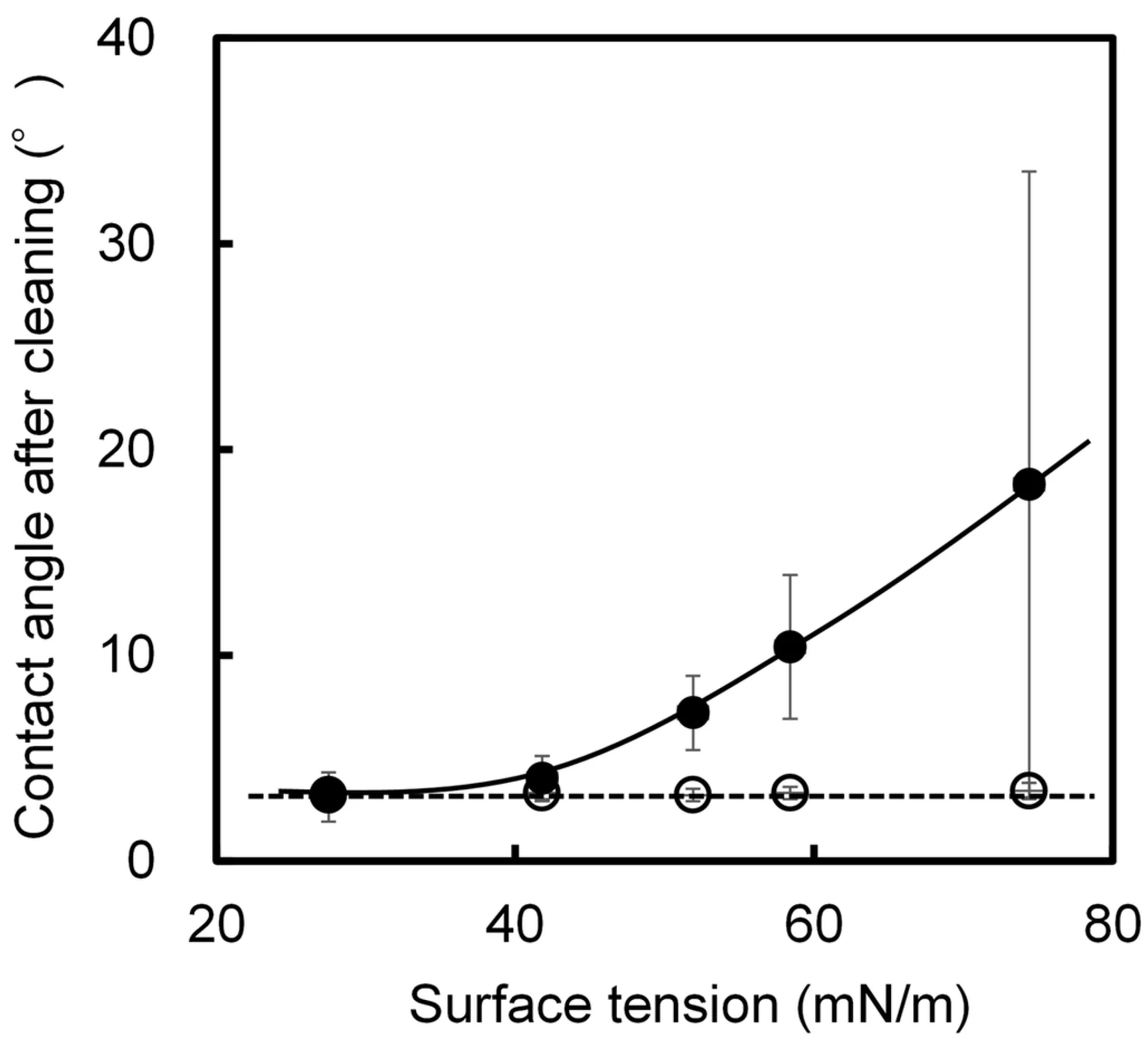
Change in the contact angle after cleaning with respect to the surface tension of the cleaning solution. Contact angle before cleaning: 20.1° (outside the TGV), 53.4° (inside the TGV). ●: inside the TGV, ◯: outside the TGV.

The effect of surface tension of the cleaning solution on the cleanability of the TGV glass was investigated. Figure 4 shows the change in the contact angle after cleaning with respect to the surface tension of the cleaning solution. Note that the contact angle outside the TGV was 20.1° and the contact angle inside the TGV was 53.4° before cleaning. As shown in Figure 4, the contact angle outside the TGV after cleaning was 3–4° under all the experimental conditions, regardless of the change in surface tension. This is presumed to be because contaminants on the glass surface were removed by the degreasing effect of the alkaline cleaning solution and the cleaning effect of the surfactants. This suggests that the surface tension of the cleaning solution had minimal effect on the cleanability outside the TGV under this condition. In contrast, the contact angle inside the TGV after cleaning tended to decrease with decreasing surface tension. At surface tensions of 27.5 and 41.8 mN/m, the contact angle inside the TGV after cleaning decreased to 3–4° and was nearly the same under both conditions, reaching a level comparable to the contact angle obtained outside the TGV. Because all surfactant-containing cleaning solutions were prepared at concentrations above their respective CMCs, the nearly identical contact angles obtained at surface tensions of 27.5 and 41.8 mN/m cannot be attributed to a transition from below to above the CMC. These results suggest that the surfactant-containing cleaning solutions with lower surface tension exhibited high cleanability inside the hydrophobized TGV. This may be related to improved wettability and dynamic wetting associated with surfactant addition, which may have shortened the penetration time of the cleaning solution into the TGV. As discussed using Equation (1), surfactant addition may decrease the contact angle between the cleaning solution and the glass surface by changing the liquid surface tension and the solid–liquid interfacial tension. This is considered to have increased the wettability of the cleaning solution and promoted its penetration into the TGV. In addition, the cleaning process inside the TGV is considered to consist of two processes: the process by which the cleaning solution penetrates into the TGV and the process by which cleaning occurs through contact of the cleaning solution with the glass surface. Within the allotted time for the cleaning process, shortening the time required for the cleaning solution to penetrate into the TGV increases the time available for cleaning. That is, it is presumed that, for cleaning solutions with lower surface tension, the degreasing effect of the alkaline cleaning solution and the cleaning effect of the surfactants act on the glass surface for a longer time, thereby promoting contaminant removal and decreasing the contact angle inside the TGV after cleaning. Taken together, these findings indicate that, under the present conditions, the surface tension of the cleaning solution is one of the factors affecting cleanability inside the TGV. Although the cleaning solution was displaced by deionized water during rinsing, residual surfactants on the glass surface were not directly quantified. Therefore, the possibility that trace surfactant residues affected the measured contact angle cannot be completely excluded. Nevertheless, the use of a cleaning solution with surface tension reduced by surfactant addition may be effective for cleaning glass substrates with fine TGVs.

### 3.2. Effect of Cleaning on Plating Process of φ20 μm TGV Glass

**Figure 5:**
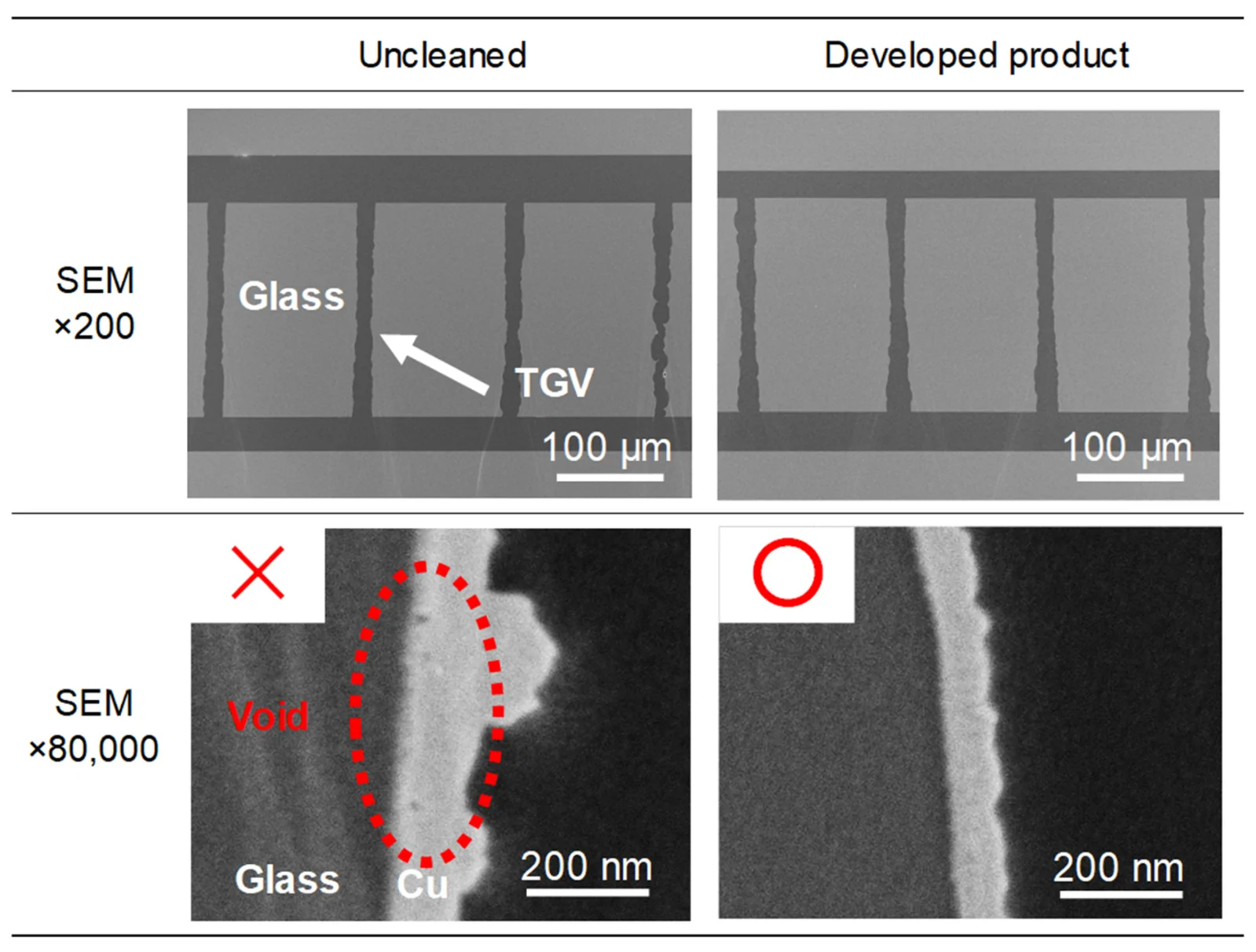
Cross-sectional SEM images of TGVs in the *φ*20 μm TGV glass subjected to electroless Cu plating under each cleaning condition. Evaluation criteria: ◯, no defects; ×, defects present.

The effect of cleaning on the electroless Cu plating process of the *φ*20 μm TGV glass was then investigated. Figure 5 shows the cross-sectional SEM images of TGVs in the *φ*20 μm TGV glass subjected to electroless Cu plating under each cleaning condition. In Figure 5, the 200× SEM images show the TGV structure, while the 80,000× SEM images show the glass/Cu interface near the center of the TGV. The 80,000× SEM image shows the formation of voids in the Cu film under the uncleaned condition. This is presumably because a nonuniform plating reaction occurred due to contaminants on the glass surface inside the TGV. In comparison, the 80,000× SEM image under the condition cleaned with the developed product did not show void formation in the Cu film. This is presumably because the removal of contaminants from the glass surface inside the TGV by cleaning reduced the impact of contaminants on Pd catalyst application and electroless plating. These observations suggest that, under the present conditions, cleaning with an alkaline solution having a surface tension of 27.5 mN/m may be effective in reducing void formation in the Cu film inside the TGVs in the *φ*20 μm glass.

**Figure 6:**
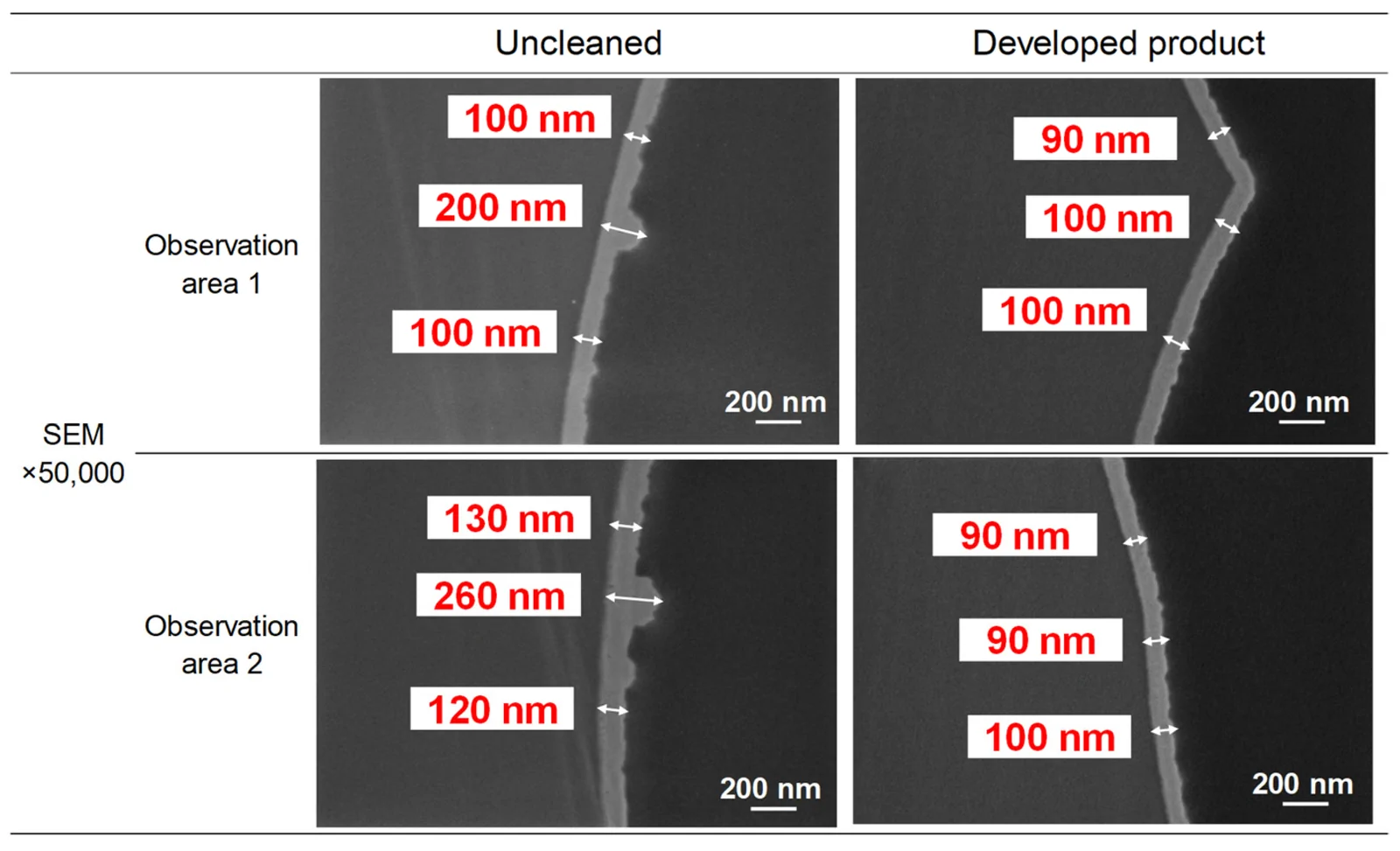
Results of film thickness measurements inside the TGV in the *φ*20 μm TGV glass under each cleaning condition.

As shown in Figure 5, a difference in the thickness of the Cu film (hereinafter referred to as the film thickness) was observed between the uncleaned condition and the condition cleaned with the developed product. Therefore, the film thickness was measured from the SEM observations. Figure 6 shows the results of the film thickness measurements inside the TGV in the *φ*20 μm TGV glass under each cleaning condition. SEM observations were performed at a magnification of 50,000×. For each condition, images were acquired at two locations, and the film thickness was measured at three representative points in each image. As shown in Figure 6, under the uncleaned condition, the film thickness was in the range 100–260 nm and was nonuniform. This is presumably because a nonuniform plating reaction occurred due to contaminants on the glass surface inside the TGV. In contrast, under the condition cleaned with the developed product, the film thickness was in the range of 90–100 nm and was more uniform. This is presumably because a more uniform plating reaction may have occurred owing to the removal of contaminants from the glass surface inside the TGV by cleaning. Because the electroless Cu plating experiment was performed once for each condition, the SEM images and film-thickness measurements should be regarded as representative observations, and no statistical generality is claimed. Nevertheless, these observations suggest that, under the present conditions, removing contaminants from the glass surface by cleaning may contribute to the formation of a Cu film with a more uniform thickness.

### 3.3. Penetration Time of the Cleaning Solution into TGVs in the φ20 μm TGV Glass

Within a given cleaning time, a shorter penetration time of the cleaning solution into the TGV is expected to increase the effective cleaning time inside the TGV. Therefore, to examine the penetration time of the cleaning solution into the TGVs in the *φ*20 μm glass, the penetration time was measured by dropping an aqueous NaOH solution and the developed product as representative cleaning solutions onto the TGV formation areas in the *φ*20 μm glass. Table 3 presents the surface tension of each cleaning solution and the penetration time into the *φ*20 μm TGVs. As listed in Table 3, the NaOH solution did not penetrate for more than 600 s after dropping. In contrast, the developed product penetrated 210 s after dropping. As the *φ*20 μm TGV glass used in this test was not pre-cleaned, it was inferred that the internal surface of the TGV had become hydrophobic. Therefore, as the NaOH solution, which does not contain a surfactant, is unlikely to wet the hydrophobic glass surface inside the TGV, it is considered that the contact angle between the NaOH solution and the glass surface is high. In other words, it is presumed that the capillary pressure driving penetration into the TGV was low, and therefore penetration did not occur within 600 s. In contrast, the developed product may have exhibited better wettability on the hydrophobic glass surface inside the TGV due to the action of the surfactant; therefore, the contact angle between the developed product and the glass surface was considered to be low. Furthermore, the shorter penetration time compared with that of the NaOH solution is consistent with the possibility that penetration into the TGV was promoted by the dynamic wetting effect of the surfactant superimposed on capillary pressure. It should be noted that Equation (2) is based on a simplified cylindrical capillary model and does not account for the variation in diameter of the hourglass-shaped TGVs used in this study. In the region extending from the TGV opening to the minimum-diameter region, the magnitude of the capillary pressure may increase as the radius decreases, while the viscous resistance may simultaneously increase. After the liquid passes through the minimum-diameter region and enters the diverging region, the magnitude of the capillary pressure is considered to decrease as the local radius increases [47]. Therefore, the actual penetration behavior may depend on the minimum diameter, taper angle, surface condition, and contact angle. These geometric effects were not independently evaluated in this study. Because the penetration test was performed only with the NaOH solution and the developed product as representative cleaning solutions, the continuous relationship between surface tension and penetration time was not evaluated. Despite these limitations, the results showed that, under the present conditions, the developed product, which contained surfactants and had a surface tension of 27.5 mN/m, penetrated the *φ*20 μm TGV within 210 s, whereas the NaOH solution did not penetrate within 600 s. The shorter penetration time of the cleaning solution may have contributed to more effective cleaning inside the TGV. These results suggest that surfactant addition may be effective for improving cleanability inside the hydrophobized TGV.

## 4. Conclusions

In this study, the influence of the surface tension of the cleaning solution on the cleanability of TGV glass was examined using Eagle XG glass containing hourglass-shaped TGVs as an example of TGV glass, based on the contact angle between the glass surface and water as an indirect measure. Under the tested conditions, the surface tension of the cleaning solution had minimal influence on the cleanability outside the TGV. In contrast, in terms of the cleanability inside the TGV, the contact angle after cleaning decreased with a decrease in the surface tension of the cleaning solution. This result indicated that, under the present conditions, the surface tension of the cleaning solution was one of the factors affecting the cleanability inside the TGV. Furthermore, under the present conditions, cleaning with a cleaning solution having a surface tension of 27.5 mN/m was associated with reduced void formation in the Cu film inside the TGVs in the *φ*20 μm glass and with improved uniformity of the Cu film thickness. This cleaning solution, which contained the surfactants, required a shorter time to penetrate the uncleaned *φ*20 μm TGV than the NaOH solution.

Our findings suggest that, under the present conditions, the addition of the surfactant and reduction in the surface tension of the cleaning solution may help alleviate cleaning issues inside TGVs hydrophobized by contamination. The TGV fabrication method and inner-wall roughness were not disclosed by the supplier. These factors may affect the surface condition and penetration behavior. Therefore, the present findings are limited to the Eagle XG substrates and the TGV geometries shown in Figure 1 and should not be directly generalized to other glass materials or TGV structures. These findings suggest that cleaning TGV glass may be an important factor in reducing defects in the Cu film inside the TGV and may support the practical use of TGV glass substrates for advanced packaging.

## Figures and Tables

**Table 1:** Components and properties of the cleaning solutions used in the tests.

	NaOH Solution	ST60	ST50	ST40	Developed Product
Components		Water (99.0–99.7 wt%)	Water (99.0–99.7 wt%)	Water (99.0–99.7 wt%)	Water (99.0–99.7 wt%)
	Water (95 wt%)	KOH (0.24 wt%)	KOH (0.24 wt%)	KOH (0.24 wt%)	KOH (0.24 wt%)
	NaOH (5 wt%)	Surfactant (0.05–0.5 wt%)	Surfactant (0.05–0.5 wt%)	Surfactant (0.05–0.5 wt%)	Surfactant (0.05–0.5 wt%)
		Additive (0.01–0.2 wt%)	Additive (0.01–0.2 wt%)	Additive (0.01–0.2 wt%)	Additive (0.01–0.2 wt%)
pH (25 °C)	13.6	12.5	12.6	12.5	12.6
Surface tension (mN/m)	74.4	58.4	51.9	41.8	27.5

**Table 2:** Electroless Cu plating process of the *φ*20 μm glass.

Process	Chemical Solution	Method	Temperature	Time
Cleaning	Developed product	Ultrasonic immersion	50 °C	1 min
Rinsing	Deionized water	Three-stage overflow rinsing Flow rate: 4 L/min	Room temperature	1 min
Pd catalyst addition	Not disclosed due to contract	Ultrasonic immersion	Room temperature	1 min
Rinsing	Deionized water	Three-stage rinsing	Room temperature	-
Electroless Cu plating	Not disclosed due to contract	Ultrasonic immersion	60–75 °C	8 min
Rinsing	Deionized water	Three-stage rinsing	Room temperature	-
Drying	-	Hot air drying	-	-

**Table 3:** Surface tension of each cleaning solution and penetration time into the *φ*20 μm TGVs.

	NaOH Solution	Developed Product
Surface tension	74.4 mN/m	27.5 mN/m
Penetration time	>600 s	210 s

## Data Availability

The original contributions presented in the study are included in the article; further inquiries can be directed to the corresponding author.
